# A dataset of hidden small non-coding RNA in the testis of heat-stressed models revealed by Pandora-seq

**DOI:** 10.1038/s41597-024-03612-6

**Published:** 2024-07-09

**Authors:** Mailin Gan, Yuhang Lei, Kai Wang, Yan Wang, Tianci Liao, Jianfeng Ma, Li Zhu, Linyuan Shen

**Affiliations:** 1 Farm Animal Genetic Resources Exploration and Innovation Key Laboratory of Sichuan Province, Sichuan Agricultural University, Chengdu, 611130 China; 2 Key Laboratory of Livestock and Poultry Multi-omics, Ministry of Agriculture and Rural Affairs, College of Animal and Technology, Sichuan Agricultural University, Chengdu, 611130 China; 3 State Key Laboratory of Swine and Poultry Breeding Industry, College of Animal Science and Technology, Sichuan Agricultural University, Chengdu, 611130 China

**Keywords:** Non-coding RNAs, Sequencing

## Abstract

Infertility, a worldwide reproductive health concern, impacts approximately one in five couples. Male infertility, stemming from spermatogenic dysfunction and reduced sperm quality, stands as a primary factor contributing to infertility. Given the global decrease in male fertility linked to environmental factors like the greenhouse effect, it is crucial to develop a comprehensive understanding of how increased temperatures impact both the quantity and quality of sperm. In this study, we utilized Pandora-seq technology to detect the small non-coding RNAs (sncRNAs) expression profile in the testicular tissue of heat-stressed mice. The investigation explores the dynamic shifts in sncRNAs within the mouse testis under heat stress, including miRNAs, tsRNAs, piRNAs, rsRNAs and other sncRNAs. Furthermore, we successfully identified differentially expressed sncRNAs in testicular tissues before and after heat stress. Subsequently, we conducted functional enrichment analysis on the potential predicted target genes of differentially expressed miRNAs and tsRNAs. These datasets will constitute a valuable foundational resource for further investigations into the decline in male reproductive capacity triggered by heat stress.

## Background & Summary

Infertility, namely reproductive failure, has become the main problem of human reproductive health. It is estimated that infertility affects up to one-fifth of couples worldwide^1^. Among them, infertility caused by male factors accounts for 30–50% of infertility cases^2,3^. Given the established correlation between semen quality and successful pregnancy, the evaluation of male reproductive capacity often relies on semen quality assessments^4–6^. Notably, global male fertility is declining rapidly, with average sperm count and concentration dropping by approximately 50%^7^. Studies have indicated that the greenhouse effect is considered to be the current culprit for the reduction of semen quality in men^8,9^. Scrotal temperature profoundly impacts male reproductive health and semen quality. Usually, maintaining testicular temperature 2–4 °C lower than the core body temperature is crucial for optimal spermatogenesis. However, when the testis is exposed to heat stress, it accelerates germ cell apoptosis^10^. The evidence has revealed that for every 1 °C rise in temperature, there’s a correlated decrease in sperm production by 14%, significantly impacting overall sperm count^11^. Hence, it is imperative to comprehend the underlying causes behind the decline in both quality and quantity of male sperm due to high temperatures. This understanding holds significant importance in averting the decline in male fertility.

**Fig. 1:**
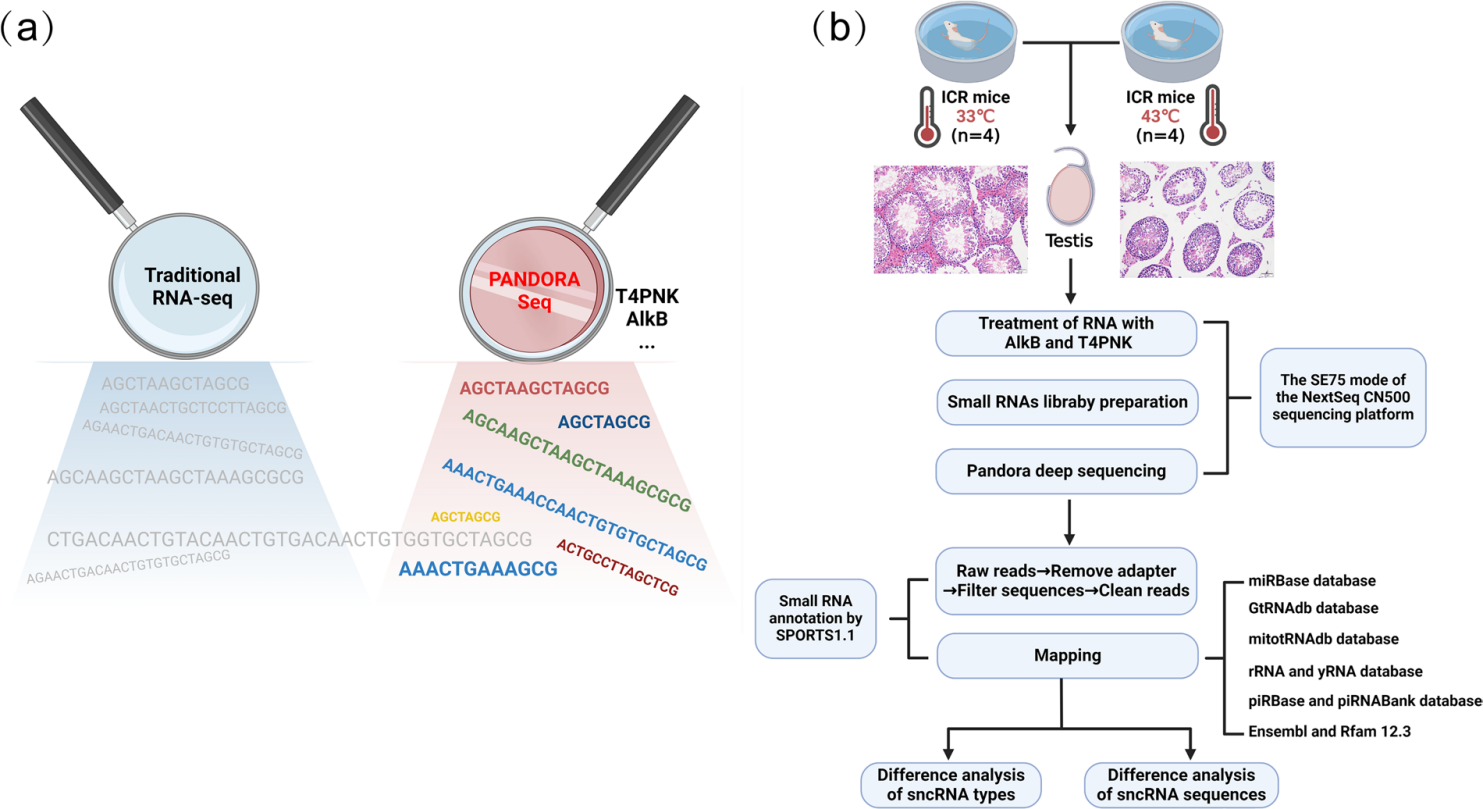
Overview of the experimental process. (**a**) Illustrations of comparison between Pandora-seq and RNA-seq. (**b**) Illustrations of mouse model construction, sample collection, and Pandora sequencing experimental procedures.

In recent years, there has been a noticeable increase in research attention directed towards understanding the involvement of non-coding RNAs across diverse biological processes^12^. Non-coding RNAs include long non-coding RNAs (lncRNAs) and small non-coding RNAs (sncRNAs). Over the past decade, high-throughput RNA sequencing technology has significantly advanced the exploration of functional sncRNAs, notably miRNAs. However, when sncRNAs carry specific modifications, including 3′end modification (blocking the adapter ligation process) and RNA methylation modification (interfering with the reverse transcription process), the conversion efficiency of sncRNAs to cDNA is greatly reduced, resulting in reduced sequencing depth and accuracy^13,14^. To address these challenges, a novel RNA-Seq approach named Pandora-Seq (Panoramic RNA Display by Overcoming RNA modification aborted sequencing) has been developed, effectively circumventing the interference of RNA modifications with sequencing results^14^. The sequencing approach relies on enzymatic treatment of small RNAs within the 15–50 nucleotide range, employing dealkylase α-ketoglutarate-dependent hydroxylase (AlkB) and its mutant form^15^, as well as T4 polynucleotide kinase treatment^16,17^. This process effectively eliminates sequencing-affecting modifications, resulting in a comprehensive profile of sncRNAs (Fig. 1a).

Epigenetic changes, including alterations in sncRNAs, play a pivotal role in gametogenesis and are closely linked to various reproductive disorders^18^. Recognized as pivotal contributors to epigenetic regulation, sncRNAs have garnered considerable attention for their crucial involvement in testicular development and spermatogenesis^19–21^. In the context of global warming induced by the greenhouse effect, heat stress factors are impacting male semen quality and fertility^8^. Hot water bath is a common form of testicular heat stress. Typically, exposure to hot water for over 30 minutes per day can lead to specific damage in the anatomical morphology and sperm quality of the male testis within a week^22^. Previous studies predominantly delved into specific types of sncRNAs, such as microRNA^23^ or piRNA^24^, to understand testicular damage induced by heat stress. However, the dynamic alterations within the intricate regulatory network of sncRNAs in the testis under heat stress remain unexplored. Therefore, to reveal the dynamic changes of the sncRNA library in the mouse testis under heat stress, we collected testicular tissues from both the normal group (NC) and hot water group (HW) mice, obtaining their Pandora sequencing data (Fig. 1b). We analyzed a total of 8 samples, with 4 biological replicates in NC and HW group. Details of the specific samples are presented in Table 1. We used the Illumina NextSeq sequencing platform to generate a total of 278676235 raw reads. The sequencing results were annotated using the small RNA annotation software SPORTS1.1, yielding a total of 99,192,693 match genome reads (Table 2). Our data comprehensively delineate the dynamic changes in small non-coding RNAs (sncRNAs) in testicular tissue before and after heat stress in mice, including miRNAs, tsRNAs, piRNAs, rsRNAs and other sncRNAs.

**Table 1:** Basic sample collection information of mice.

Sample	Age	Weight (g)	Testicular weights (Left, g)	Testicular weights (Right, g)
NC-5-T	Ten weeks	35.93	0.144	0.136
NC-6-T	Ten weeks	38.59	0.132	0.128
NC-8-T	Ten weeks	33.61	0.136	0.136
NC-9-T	Ten weeks	36.51	0.134	0.145
HW-4-7d-T	Ten weeks	36.67	0.061	0.061
HW-5-7d -T	Ten weeks	37.82	0.045	0.045
HW-9-7d -T	Ten weeks	38.36	0.065	0.065
HW-10-7d -T	Ten weeks	34.47	0.045	0.045

**Table 2:** Summary of PANDORA-Seq Reads.

Sampe_ID	Raw reads	Clean_Reads	Match_Genome_Reads	miRNA_Match_Reads	rsRNA_Match_Reads	tsRNA_Match_Reads	mt_tsRNA_Match_Reads	piRNA_Match_Reads
NC-5-T	21319881	12401143	8080074	55637	4703789	1294408	112163	216906
NC-6-T	23370251	13606239	8947771	57031	5307689	1356660	130939	226705
NC-8-T	23209913	13152939	8691264	56235	5036033	1371494	128175	243235
NC-9-T	25322716	13623797	8596144	29107	5478653	1150744	73845	168384
HW-4-7d-T	21288819	11781150	7325811	90114	4958519	1172503	125238	11860
HW-5-7d-T	20469897	11022159	7223968	84806	4851244	1079033	171800	8788
HW-9-7d-T	25126108	13647748	8076794	111492	5333673	1042828	133340	16945
HW-10-7d-T	24133041	13127170	8553828	83547	5390575	1068322	156340	14799

Raw reads: Original sequencing readings without quality filtering and clean. Clean reads: SPORTS 1.1 outputs clean reads by removing the sequence adapter, discarding reads obtained by sequences that are longer than the defined range and non-ATUCG sequences. Match Genome Reads: Reads number of raw reads aligned with the reference genome readings. miRNAs match reads: Number of reads aligned to miRNAs sequences. rsRNAs match reads: Number of reads aligned to rRNAs sequences. tsRNAs match reads: Number of reads aligned to mature and precursor tRNAs. mt-tsRNAs match reads: Number of reads aligned to mature and precursor tRNAs in mitochondria. piRNAs match reads: Number of reads aligned to PIWI-interacting RNAs sequences.

## Methods

### HW mice preparation and sample collection

In this study, we selected 8 ten-week-old male ICR mice (36.50 ± 1.79 g) from DOSSY Experimental Animals Co., Ltd. (Chengdu, China). To establish the testicular heat stress model, mice underwent a daily 25-minute immersion in 43 °C hot water for one week, while the control group experienced baths in 33 °C water. One week following the hot water bath treatment, eight ICR mice were euthanized, and their testicular and epididymal tissues were promptly collected and frozen in liquid nitrogen. The ethical considerations of this study were approved by the Ethics Committee of Sichuan Agricultural University.

### Total RNA isolation and quality control

Total RNA was extracted with TRIzol reagent according to manufacturer’s instructions (Invitrogen; 15596018). Agarose gel electrophoresis was utilized to evaluate the quality of the total RNA sample, followed by the isolation of RNA of the specified size from the total RNA. Concurrently, RNA concentration was determined using a Nanodrop spectrophotometer (NanoDrop 2000, Thermo Fisher Science, USA).

### Treatment of RNA with AlkB and T4PNK

Before constructing the small RNA-seq library and performing deep sequencing, the purified small RNA was treated with T4PNK and AlkB to address the issue of RNA modification hindering the passability of reverse transcriptase. Initially, RNA underwent treatment with AlkB enzyme. The RNA was incubated in 50 μL reaction mixture containing 50 mM HEPES (pH 8.0) (Gibco (15630080) and Alfa Aesar (J63578), 75 μM ferrous ammonium sulfate (pH 5.0), 1 mM α-ketoglutaric acid (Sigma-Aldrich; K1128-25G), 2 mM sodium ascorbate, 50 mg l^−1^ bovine serum albumin (Sigma-Aldrich; A7906-500G), 4 μg ml^−1^ AlkB (Guangzhou Epibiotek Co., Ltd., Guangzhou, China), 2,000 U RNase inhibitor and 200 ng RNA at 37 °C for 30 min. Then, the mixture was added into 500 μL TRIzol reagent to perform the RNA isolation procedure. Subsequently, RNA was subjected to treatment with T4PNK. The RNA was incubated in 50 μL reaction mixture containing 5 μL 10 × PNK buffer (New England Biolabs; B0201S), 10 mM ATP (New England Biolabs; P0756S), 10 U T4PNK (New England Biolabs; M0201L) and 200 ng RNA at 37 °C for 20 min. Then, the mixture was added into 500 μL TRIzol reagent to perform the RNA isolation procedure.

### Small RNA library construction and deep sequencin

The RNA segment was separated by PAGE, then a 15- to 45-nucleotide stripe was selected and recycled. The adapters were obtained from the QIAseq® miRNA Library Kit (QIAGEN: 331505) and ligated sequentially. The amplified flow cell was sequenced on the Illumina system by epibiotek (Guangzhou Epibiotek Co., Ltd., Guangzhou, China). The raw sequencing data were generated using the SE75 mode of the NextSeq CN500 sequencing platform.

### Small RNA annotation and analyses for PANDORA-seq data

The annotation of small RNA sequences was carried out using the non-coding RNA annotation and analysis software SPORTS1.1, specifically optimized for small RNAs derived from rRNA and tRNA. Reads were mapped to the following individual non-coding RNA databases sequentially: (1) the microRNA database miRBase 21; (2) the rRNA and YRNA databases assembled from the National Center for Biotechnology Information nucleotide and gene database; (3) the genomic tRNA database GtRNAdb; (4) the mitochondrial tRNA database mitotRNAdb; (5) the piRNA database, including piRBase and piRNABank; (6) the non-coding RNAs defined by Ensembl and Rfam 12.3. In the annotation of tsRNAs, both pre-tRNA and mature tRNA sequences were employed for comprehensive annotation. Mature tRNA sequences were derived from the GtRNAdb and mitotRNAdb sequences using the following procedures: (1) predicted introns were removed; (2) a CCA sequence was added to the 3′ ends of all tRNAs; and (3) a G nucleotide was added to the 5′ end of histidine tRNAs. The tsRNAs were categorized into four types based on the origin of the tRNA loci: (1) 5′ tsRNA (derived from the 5′ end of pre-/mature tRNA); (2) 3′ tsRNA (derived from the 3′ end of pre-tRNA); (3) 3′ tsRNA-CCA end (derived from the 3′ end of mature tRNA); (4) and internal tsRNAs (not derived from 3′ or 5′ loci of tRNA). For rsRNA annotation, rsRNAs derived from 4.5 S, 5 S, 5.8 S, 12 S, 16 S, 18 S, 28 S, and 45 S rRNA were distinguished based on the sequence fragment size of rRNA. Mapping to the parent rRNA in ascending order of rRNA sequence length will substantially ensure the uniqueness of each rsRNA annotation. For tsRNA annotation, annotation was based on pre-tRNA and mature tRNA. DESeq 2 was employed to identify sncRNAs with significant differences, using screening criteria of log_2_(Fold Change) > 1 and P < 0.05.

#### Dynamic changes of sncRNAs expression and sequence characteristics in mouse testicular tissue before and after heat stress

**Fig. 2:**
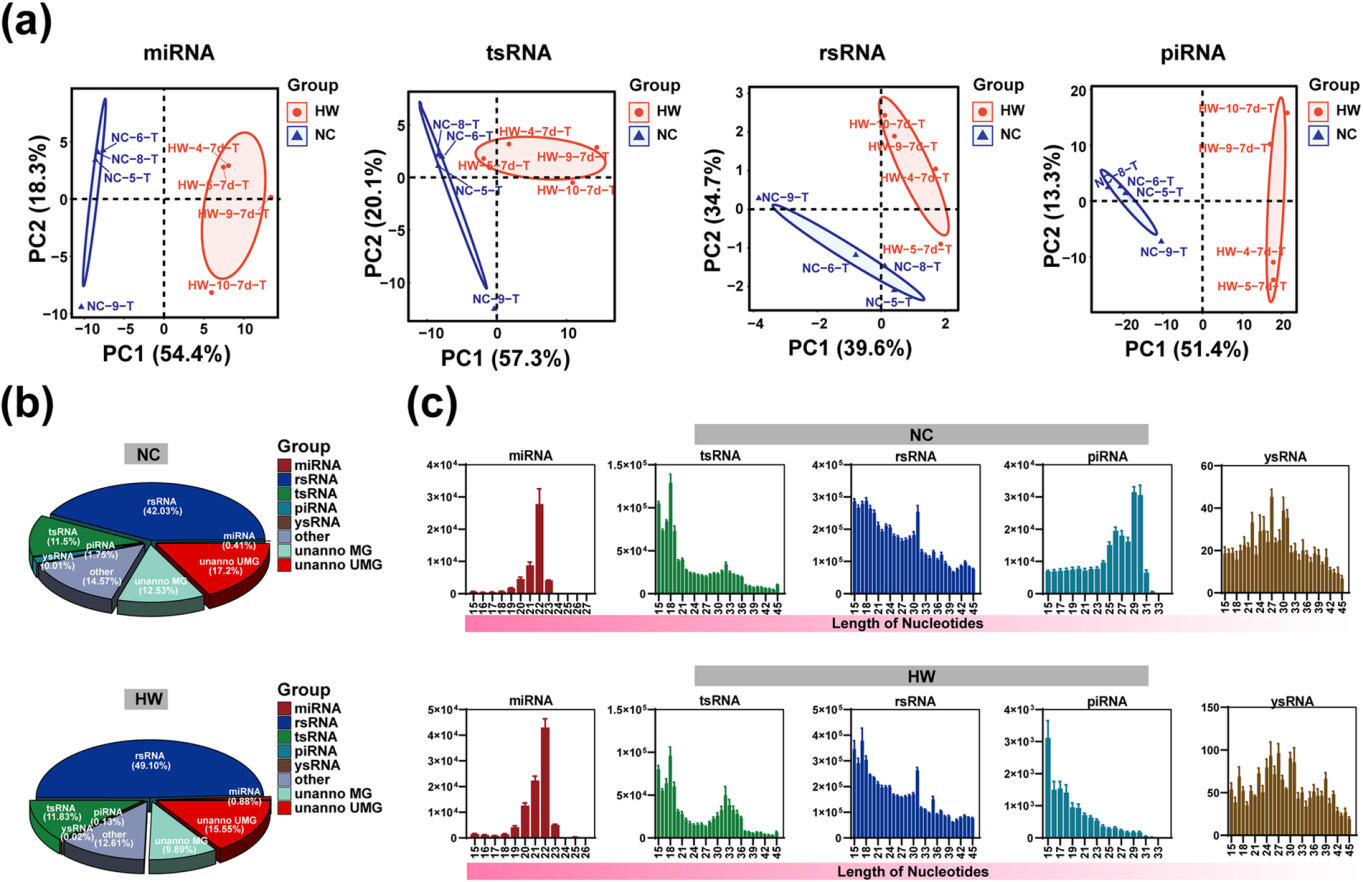
The expression and sequence characteristics of sncRNAs in testicular tissue of mice before and after heat stress. (**a**) Principal component analysis (PCA) based on the reading counts of various sncRNAs in the sample. (**b**) The distribution of sncRNAs types in samples before and after heat stress was expressed in proportion. (**c**) Length distribution of various types of sncRNAs in samples before and after heat stress.

Initially, we performed principal component analysis (PCA) on the read counts of miRNA, tsRNA, rsRNA, and piRNA for each sample (Fig. 2a). The PCA results highlighted significant differences in the distribution of testicular tissue samples before and after heat stress across each sncRNA family. By analyzing the read counts of diverse sncRNAs, we characterized the expression profile of sncRNAs in mouse testicular tissue before and after exposure to heat stress. The pie chart illustrates the proportion distribution of different types of sncRNAs in testicular tissue before and after heat stress treatment (Fig. 2b). The results revealed that upon the onset of testicular heat stress, the proportion of rsRNA expression increased from 42.03% to 49.10%, whereas the proportion of piRNA expression dropped from 1.75% to 0.13%. We further investigated the nucleic acid length distribution for each subtype of sncRNAs (Fig. 2c). Specifically, miRNA lengths were concentrated around 22 nt, tsRNA exhibited distinct peaks at 18 nt and 32 nt, rsRNA displayed a peak at 31 nt, and ysRNA presented a peak with relatively less clarity. Interestingly, the peak value of piRNA was evident at 29 nt or 30 nt before the induction of heat stress. However, upon the occurrence of heat stress, there was a noticeable reduction in the overall expression of piRNA, and the peak value became less defined.

#### The sncRNA landscape of rsRNA and tsRNA before and after heat stress in mice

**Fig. 3:**
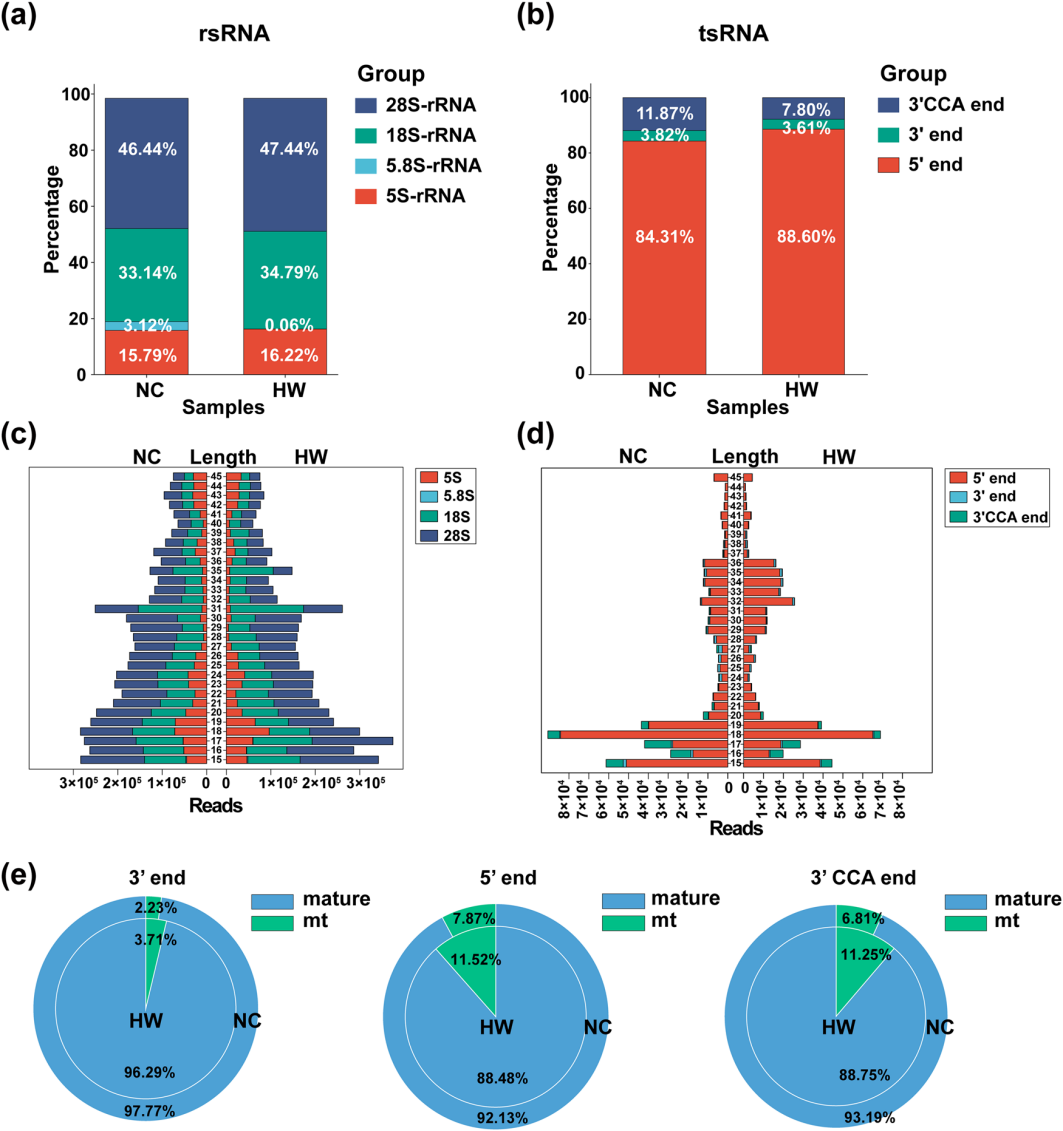
The sncRNA landscape of rsRNA and tsRNA before and after heat stress in mice. (**a**) Expression patterns of rsRNA fragments in samples before and after heat stress. (**b**) Expression patterns of tsRNA types in samples before and after heat stress. (**c**) Distribution of sequence lengths among rsRNA fragment subtypes. (**d**) Distribution of sequence lengths for tsRNA types. (**e**) Distribution of tsRNA type sources.

Taking into consideration the diverse types of rsRNA and tsRNA, we conducted a detailed analysis of the subtypes within these two categories of sncRNAs. Figure 3a illustrated the distribution of the number of rsRNAs derived from fragments of different lengths. The absolute proportion of rsRNA originating from 28 S rRNA was approximately 50% in both sample groups. Furthermore, following heat stress in mouse testis, there was a notable decrease in the proportion of rsRNA derived from 5.8 S rRNA. It is worth noting that, as previously illustrated in Fig. 2b, the proportion of rsRNA expression increased under heat stress conditions. Similarly, we analyzed the distribution of various types of tRNA derived fragments in the samples (Fig. 3b). The results indicated that heat stress treatment elevated the proportion of 5′ tsRNA in mouse testicular tissue while decreasing the proportion of 3′ CCA tsRNA. Additionally, we visualized the sequence length distribution for each type of rsRNA and tsRNA (Fig. 3c,d). We also conducted an analysis of the sources of different types of tsRNA, which include pre-tRNA, mature tRNA, and mitochondrial tRNA. Due to the exceptionally low content of pre-tRNA, it is not represented in the pie chart (Fig. 3e). Interestingly, heat stress appears to have no influence on the content of 3′ tsRNA from diverse sources in the mouse testis. However, heat stress led to a reduction in the expression of 5′ tsRNA and 3′ CCA tsRNA derived from mature tRNA, while simultaneously increasing the expression of 5′ tsRNA and 3′ CCA tsRNA derived from mitochondrial tRNA.

#### Identification of differentially expressed sncRNAs

**Fig. 4:**
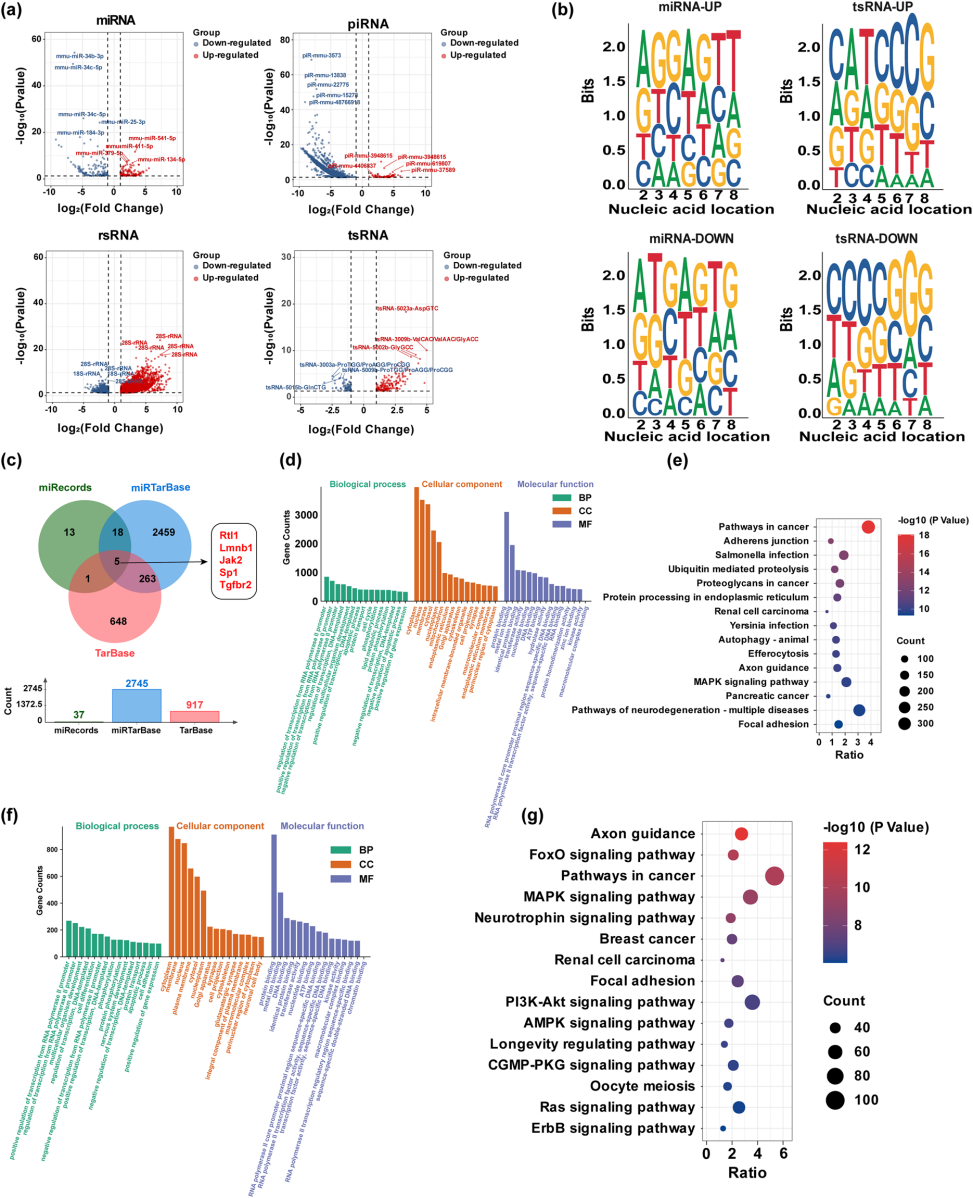
Identification of differentially expressed sncRNAs before and after heat stress in mice. (**a**) Volcano plot of differentially expressed sncRNAs between NC and HW groups. (**b**) Bias of differentially expressed miRNA and tsRNA seed sequences. (**c**) Venn diagram of potential target genes predicted by differentially expressed miRNAs in three databases. (**d**) GO enrichment analysis of potential target genes of miRNAs. Each type showed the enrichment results of the top 15 of gene count. (**e**) KEGG analysis of potential target genes of miRNAs. Only the top 15 signaling pathways with the most significant differences were showed. (**f**) GO enrichment analysis of potential target genes of tsRNAs. Each type showed the enrichment results of the top 15 of gene count. (**g**) KEGG analysis of potential target genes of tsRNAs. Only the top 15 signaling pathways with the most significant differences were showed.

Differentially expressed sncRNA analysis was performed using the R package DESeq 2. The criteria used for screening differentially expressed sncRNAs were log_2_(Fold Change) > 1 and P value < 0.05. We visualized the differentially expressed sncRNAs based on the screening conditions. The red circle represents up-regulated sncRNAs in the HW group, while the blue circle represents down-regulated sncRNAs (Fig. 4a). Simultaneously, utilizing P values as a criterion, we identified and labeled the top five up-regulated and down-regulated sncRNAs with the most significant differences. Given that miRNA and tsRNA exert their functions relying on the 5′-end seed sequence, we conducted an analysis of the base preference of the seed sequence in miRNA and tsRNA (Fig. 4b). We observed a significant difference in the base site preference of seed sequences for differentially expressed miRNAs and tsRNAs between the two sample groups before and after heat stress. For differentially expressed miRNAs (Threshold: log_2_FC > 1.5 and P value < 0.05), we predicted their potential target genes using three databases: miRecords, miRTarBase, and TarBase (https://jingege.shinyapps.io/jingle_molecular/). A total of 3408 validated target genes were predicted, and five of these target genes were consistently displayed in all three databases (Fig. 4c). For potential target genes of miRNAs, we conducted GO and KEGG functional enrichment analysis using the DAVID website (https://david.ncifcrf.gov/summary.jsp). GO functional enrichment analysis revealed that the potential target genes of miRNAs were predominantly enriched in biological processes such as transcriptional regulation, cell survival and death, and protein function and transport (Fig. 4d). KEGG analysis demonstrated that the potential target genes of differentially expressed miRNAs, both before and after heat stress, were predominantly enriched in pathways related to signal transduction, cell survival, and metabolism (Fig. 4e). Similarly, for differentially expressed tsRNAs, we predicted their potential target genes using the online prediction website TargetScan (https://www.targetscan.org/vert_50/seedmatch.html) and conducted GO and KEGG functional enrichment analysis using the DAVID website (https://david.ncifcrf.gov/summary.jsp). GO functional enrichment analysis revealed that the potential target genes of tsRNA were predominantly enriched in biological processes such as transcriptional regulation, cell development and differentiation, and cell survival and death (Fig. 4f). KEGG analysis showed that the potential target genes of differentially expressed tsrna before and after heat stress were mainly enriched in pathways related to signal transduction, cytoskeleton and signal regulation, and cell metabolism (Fig. 4g).

## Data Records

The raw sequence data reported in this paper have been deposited in the Genome Sequence Archive (Genomics, Proteomics & Bioinformatics 2021) in National Genomics Data Center (Nucleic Acids Res 2022), China National Center for Bioinformation/Beijing Institute of Genomics, Chinese Academy of Sciences (GSA: CRA014959) that are publicly accessible at https://ngdc.cncb.ac.cn/gsa^25^.

## Technical Validation

### RNA quality control

**Fig. 5:**
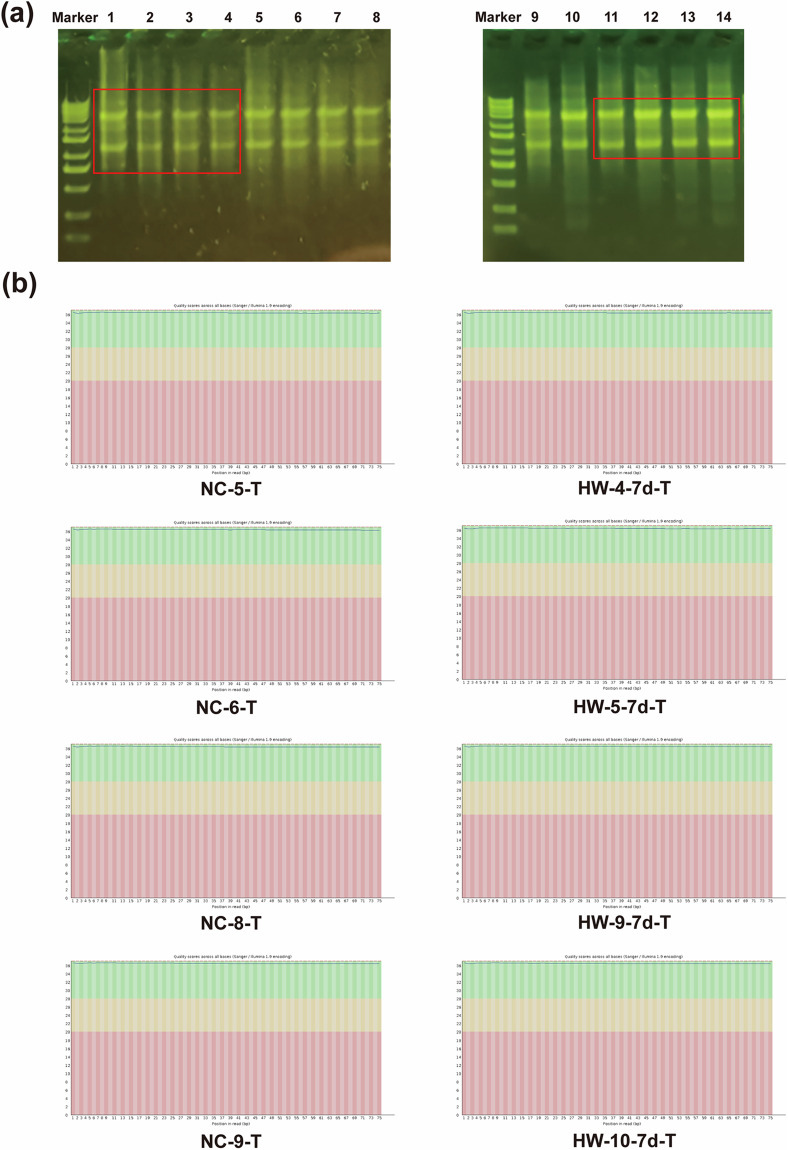
RNA agarose gel electrophoresis results and Pandora sequencing data quality control chart. (**a**) Agarose gel electrophoresis: Lanes 1–4 represent samples NC-5-T, NC-6-T, NC-8-T, NC-9-T, and lanes 11–14 represent samples HW-5-7d-T, HW-4-7d-T, HW-9-7d-T, HW-10-7d-T, respectively. (**b**) The X-axis represents the sequencing position of the base, and the Y-axis represents the sequencing quality score. The yellow area signifies a 1% error reading rate, and the green area represents a 0.1% error reading rate, indicating high data quality.

The purity and size of the extracted RNA were assessed using agarose gel electrophoresis to ensure the integrity of the samples utilized for constructing both the small RNA library and Pandora sequencing. The detailed information for each RNA sample was presented in Table 3, and the original agarose gel electrophoresis results were provided in Fig. 5a. The results indicated the good quality of our RNA samples, and the electrophoresis conditions were deemed suitable, ensuring the reliability of subsequent Pandora sequencing samples.

**Table 3:** Sample RNA quality test results.

Sample	Concentration (ng/μL)	Volume (μL)	Total volume (ng)	Quality inspection results
NC-5-T	8160	40	326400	Qualified
NC-6-T	7320	40	292800	Qualified
NC-8-T	7320	40	292800	Qualified
NC-9-T	7560	40	302400	Qualified
HW-4-7d-T	4260	40	170400	Qualified
HW-5-7d-T	3600	40	144000	Qualified
HW-9-7d-T	1584	40	63360	Qualified
HW-10-7d-T	2790	40	111600	Qualified

### Sequencing quality control

The raw sequencing data in FastQC format were obtained from the Illumina sequencing platform. Quality assessment of the sequencing data was performed using FastQC software (version 0.11.8). We created a per-base sequence quality distribution map for each sample. The y-axis represents the sequencing quality score (expressed as Q), with Q = 20% indicating an error read rate of 1%, and Q = 30% indicating an error read rate of 0.1%. The results indicated that Q > 30%, signifying good quality of the sequencing data (Fig. 5b).

## Reproducibility Validation

**Fig. 6:**
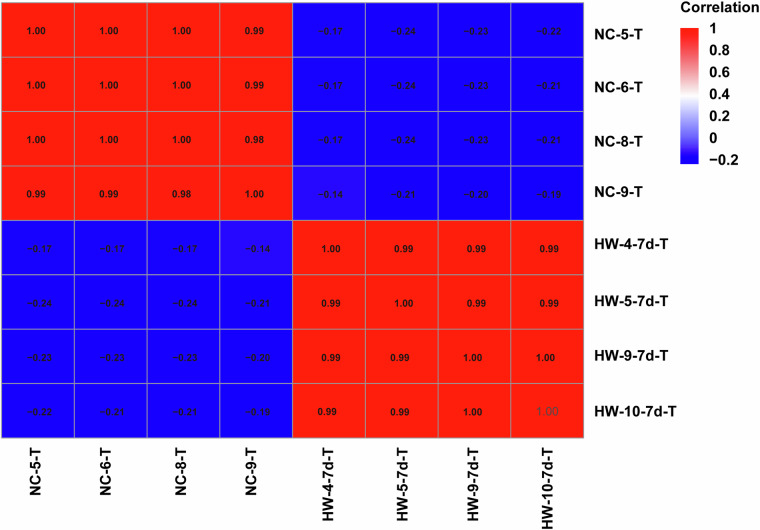
Correlation heat map of mouse testicular samples before and after heat stress.

To verify the biological repeatability of mouse testicular tissue samples before and after heat stress, we performed Pearson correlation coefficient analysis on all 8 samples. The correlation heatmap indicated a high correlation coefficient among biological replicates for both before and after heat stress samples (Fig. 6). Furthermore, principal component analysis (PCA) demonstrated distinct separation between samples before and after heat stress, with biological samples from the same treatment forming clearly clustered groups (Fig. 2a). These results affirm the high confidence and reliability of our research data.

## Data Availability

In this study, open-source bioinformatics analysis software was employed to analyze the raw sequencing data. Additionally, custom scripts or code were not utilized for dataset management and validation. Details of all software toolkits used are as follows: FastQC (version 0.11.8) was utilized for assessing the quality of the raw sequencing data (https://www.bioinformatics.babraham.ac.uk/projects/fastqc/). Bioinformatics provides an online analysis tool for principal component analysis (PCA), correlation analysis, pie charts, histogram pyramid stacks, and other functionalities (www.bioinformatics.com). The seed sequence motif was done using the Hiplot (ORG) online tool (https://hiplot.cn/). Online prediction websites, including miRecords^26^, miRTarBase^27^, and TarBase^28^ were employed to predict potential target genes of miRNAs. The potential target gene prediction of tsRNA was performed on the online prediction website Targetscan (https://www.targetscan.org/vert_50/seedmatch.html). DAVID online analysis website was employed for Gene Ontology (GO) and Kyoto Encyclopedia of Genes and Genomes (KEGG) functional enrichment analysis (https://david.ncifcrf.gov/summary.jsp). R software was used for differential expression analysis of differentially expressed sncRNAs (https://www.r-project.org/). GraphPad Prism (version 8.0.2, GraphPad Software Inc., USA) was used for statistical analyses and data visualization.
