# Author Correction: A dataset of hidden small non-coding RNA in the testis of heat-stressed models revealed by Pandora-seq

**DOI:** 10.1038/s41597-025-05008-6

**Published:** 2025-04-22

**Authors:** Mailin Gan, Yuhang Lei, Kai Wang, Yan Wang, Tianci Liao, Jianfeng Ma, Li Zhu, Linyuan Shen

**Affiliations:** 1 Farm Animal Genetic Resources Exploration and Innovation Key Laboratory of Sichuan Province, Sichuan Agricultural University, Chengdu, 611130 China; 2 Key Laboratory of Livestock and Poultry Multi-omics, Ministry of Agriculture and Rural Affairs, College of Animal and Technology, Sichuan Agricultural University, Chengdu, 611130 China; 3 State Key Laboratory of Swine and Poultry Breeding Industry, College of Animal Science and Technology, Sichuan Agricultural University, Chengdu, 611130 China

**Keywords:** Non-coding RNAs, Sequencing, Non-coding RNAs, Sequencing

Correction to: *Scientific Data* 10.1038/s41597-024-03612-6, published online 09 July 2024

In this article the figure caption for figure 4 incorrectly read ‘Conserved tsRNAs in adipose tissue of pigs and mice’ and should have been ‘Identification of differentially expressed sncRNAs before and after heat stress in mice’. The original article has been corrected.

